# Artificial Intelligence in Pediatric Emergency Medicine: Applications, Challenges, and Future Perspectives

**DOI:** 10.3390/biomedicines12061220

**Published:** 2024-05-30

**Authors:** Lorenzo Di Sarno, Anya Caroselli, Giovanna Tonin, Benedetta Graglia, Valeria Pansini, Francesco Andrea Causio, Antonio Gatto, Antonio Chiaretti

**Affiliations:** 1 Department of Pediatrics, Fondazione Policlinico Universitario “A. Gemelli” IRCCS, Università Cattolica del Sacro Cuore, 00168 Rome, Italy; anya0196@gmail.com (A.C.); benedetta.graglia@gmail.com (B.G.); antonio.chiaretti@policlinicogemelli.it (A.C.); 2 The Italian Society of Artificial Intelligence in Medicine (SIIAM), 00165 Rome, Italy; francescoandrea.causio@unicatt.it (F.A.C.); antonio.gatto@policlinicogemelli.it (A.G.); 3 Department of Pediatrics, Fondazione Policlinico Universitario “A. Gemelli” IRCCS, 00168 Rome, Italy; giovannatoninp@gmail.com (G.T.); pansini.valeria@gmail.com (V.P.); 4 Section of Hygiene and Public Health, Department of Life Sciences and Public Health, Università Cattolica del Sacro Cuore, 00168 Rome, Italy

**Keywords:** artificial intelligence, machine learning, pediatrics, deep learning, pediatric emergency medicine

## Abstract

The dawn of Artificial intelligence (AI) in healthcare stands as a milestone in medical innovation. Different medical fields are heavily involved, and pediatric emergency medicine is no exception. We conducted a narrative review structured in two parts. The first part explores the theoretical principles of AI, providing all the necessary background to feel confident with these new state-of-the-art tools. The second part presents an informative analysis of AI models in pediatric emergencies. We examined PubMed and Cochrane Library from inception up to April 2024. Key applications include triage optimization, predictive models for traumatic brain injury assessment, and computerized sepsis prediction systems. In each of these domains, AI models outperformed standard methods. The main barriers to a widespread adoption include technological challenges, but also ethical issues, age-related differences in data interpretation, and the paucity of comprehensive datasets in the pediatric context. Future feasible research directions should address the validation of models through prospective datasets with more numerous sample sizes of patients. Furthermore, our analysis shows that it is essential to tailor AI algorithms to specific medical needs. This requires a close partnership between clinicians and developers. Building a shared knowledge platform is therefore a key step.

## 1. Introduction

Artificial intelligence (AI), once considered a distant, futuristic project, has gradually become a real-world approach in a wide range of medical fields, including pediatric emergency medicine [1,2,3].

The innovative concept of machines capable of autonomously elaborating and processing information dates back to the 50s [4]. Over time, medicine has progressively been recognized as a breeding ground for AI [2]. Nowadays, the latest generation of AI can use large datasets to analyze interactions between the included variables and develop predictions with several use cases in healthcare [2,5].

Since standard clinical decision making often relies on strict flowcharts and classifications with a well-structured approach, these new AI tools could induce a significant shift from conventional methods [3,6]. Even though effective in many cases, traditional models tend to oversimplify, occasionally missing the complexity of medical conditions. AI, through big data processing, is moving from broad categories to detailed and “point-like” classes in healthcare, ultimately tailoring the diagnosis and care pathway to the patients’ needs and ultimately fostering a more personalized medicine [7,8]. However, the development and deployment of AI solutions requires a drastic shift. AI training requires a significant amount of data, readily available and machine readable. This can slow down the adoption of AI solutions in facilities where the digitalization process is not as advanced as necessary [3]. Additionally, the introduction of new tools requires the end users, including healthcare professionals and physicians, to be equipped with knowledge and skills to embrace them. The upskilling and reskilling are a pressing issue, hindering future advances in patient care and improvements in health outcomes. Therefore, a new approach is needed to combine AI and high-quality input data [9]. This narrative review aims to provide a conceptual introduction to AI and raise awareness of its emerging clinical tools and potential applications in pediatric emergency medicine.

## 2. Materials and Methods

We designed a narrative review of the literature. The first part explores the theoretical principles of AI, providing all the necessary background to feel confident with these new state-of-the-art tools. The second part presents an informative analysis of AI models in pediatric emergencies, pointing out the actual applications and challenges until future feasible research perspectives. We examined the following bibliographic electronic databases: PubMed and the Cochrane Library, from inception date until April 2024. The search was limited to English-language papers that focused on AI in pediatric emergency medicine. The key words used for the search across electronic databases were “Artificial Intelligence” or “Machine Learning” and “Pediatric Emergency” or “Triage” or “Pediatric Sepsis” or “Traumatic brain injury”.

Each selected paper was reviewed and analyzed in full text by two authors (L.D.S. and A. Caroselli) and any discrepancies among them were solved by debate. Due to the heterogeneity of the articles examined, we focused on a qualitative analysis.

## 3. Artificial Intelligence and Subfields

Artificial intelligence (AI) is the field of study that focuses on how computers learn from data and on the development of algorithms that enable this learning [3].

AI involves numerous applications capable of processing information in non-conventional ways [10]. “Big data” is a term that was introduced in the 1990s to include datasets too large to be managed by common software [11,12]. The vast amount of information about patients’ health in massive digital archives is the source of big data in healthcare. As a matter of fact in recent years there has been a progressive trend from paper-based to digitized data [13,14]. 

Big data in healthcare can be characterized by up to six main features, the so called “6 Vs”, according to different authors:

- Volume: the continuous and exponentially incremental flow of data spanning from personal medical records up to 3D imaging, genomics, and biometric sensor readings ought to be carefully managed [13]. Innovations in data management, such as virtualization and cloud computing, are enabling healthcare organizations to store and manipulate large amounts of data more efficiently and cost-effectively [15];
- Velocity: the prompt and rapid transmission of data are a pivotal item nowadays, especially in scenarios like trauma monitoring, anesthesia in operating rooms, and bedside heart monitoring, where timely data analysis can be life-saving [13]. Besides, future applications, such as early infection detection and targeted treatments based upon real-time data, have the potential to notably decrease morbidity, mortality, and ultimately impact the outcome [15,16];
- Variety: the ability to analyze large datasets, including multimedia and unstructured formats, represents an innovation in healthcare [13]. The wide range of structured, unstructured, and semi-structured data analyzed, stands as a revolutionary change that adds complexity to healthcare data management [17]. Structured data can be easily stored, recalled, elaborated and manipulated by machinery. They come from a variety of sources, including diagnoses, medications, instrument readings, and lab values, and can be sorted into numeric or categorical fields for easy analysis [13,18]. Unstructured data are commonly generated at the point of care, including free-form text such as medical notes or discharge summaries and multimedia content such as imaging [13,18]. The main challenge is to transform this data to make it suitable for AI analysis, but this process faces some obstacles. First, adding structure to unstructured data entails healthcare providers to manually review charts or images, sort the information out and enter it into the system [19]. This makes the process slow, inefficient, and prone to bias. New powerful tools such as Natural Language Processing can speed up and streamline the information extraction process [18]. Secondly, healthcare professionals’ preference for the natural language simplicity of handwritten notes remains a major barrier to a widespread adoption of electronic health records, which require field coding at the point of care to provide structured inputs [13].
- Variability refers to the consistency of data over time [17]. Data flows are unpredictable, they change often and vary widely. It’s essential to know how to manage daily, seasonal, and event-driven data spikes [20].
- Veracity: ensuring that big data are accurate and trustworthy is critical in healthcare, where accurate information can mean the difference between life and death [13]. Nevertheless, achieving veracity faces challenges, including variable quality and difficulties in ensuring accuracy, especially with handwritten prescriptions.
- Value consists of the worth of information to various stakeholders or decision makers [21].

Big data includes clinical data sourced from Computerized Physician Order Entry (CPOE) and Clinical Decision Support (CDS) systems, as well as patient information stored in electronic patient records (EPRs), and machine-generated/sensor data, including vital sign monitoring [13]. Big data analytics may improve care and reduce costs by identifying connections and understanding patterns and trends among different items [22]. In fact, it could potentially enhance healthcare outcomes through information elaboration, healthcare provider guidance, preventative care candidates identification, and disease profiling [13]. 

In regular healthcare analytics, project analysis is typically performed using easy-to-use business intelligence tools on stand-alone systems; however, in big data analytics, the processing of large datasets is distributed across multiple nodes, requiring a shift in user interfaces [23]. While traditional analytics tools are easy to use and transparent, new tools are complex, programming intensive, and require different skill sets to be most effective.

In order to guarantee an adequate output, this huge amount of data have to be verified by valid tools. Blockchain is a technology characterized by the decentralization of entries, meaning that inputs are agreed upon by a peer-to-peer network through various consensus protocols, rather than a central authority controlling the content [24]. Furthermore, many blockchains offer anonymity or pseudo-anonymity [24,25]. Specifically for healthcare data management, these features ensure data security and privacy through a network of secure blocks linked by cryptographic protocols [24,26]. Another key feature of blockchain is persistency: once data are inserted into a block and added to the chain, it cannot be deleted [24]. This implies that if an inaccurate data are added to the blockchain, it becomes a permanent part of the ledger. Thus, it is important to ensure the accuracy of data before adding it to the blockchain [27].

The integration of AI and blockchain is promising: AI tools could leverage information acquired from a secure, unchangeable, and decentralized system for storing sensitive data required by AI-driven techniques [26]. An integration of AI and blockchain in the metaverse has been proposed in order to provide digital healthcare through realistic interactions [28]. By using blockchain for data security and privacy, healthcare providers and patients engage in consultations in a virtual environment: participants are represented by avatars, and consultation data are securely recorded and stored on the blockchain [28]. This data are then used by explainable AI models to predict and diagnose diseases, ensuring logical reasoning, trust, transparency, and interpretability in the diagnostic process [28].

Machine learning (ML), a subset of computer science and artificial intelligence, seeks to identify patterns in data to boost the effectiveness of various tasks [29]. In healthcare, ML uses automated, adaptive and computationally advanced techniques to recognize patterns within complex data structures [29]. ML models improve their performance by means of a continuous auto-training process [30,31]. This approach differs from “traditional” methods and explicit human programming, which rely on certain statistical assumptions and require a predefined set of dimensions, functional relationships, and interactions [29,31]—an issue often avoided in ML.

To develop a reliable ML model, accurate training datasets are required; therefore, a preprocessing phase is usually needed [3]. Most of the data are used to train the model with preliminary analyses performed to identify the strongest relationships between variables and study outcomes. The remaining data can be used for internal validation. At this stage, the model can be tested on different datasets [3].

ML-aided tasks have already been incorporated into clinical practice, especially in imaging interpretation [32,33,34]. Although they are still imperfect and require a skilled supervisor, they are considered acceptable when rapid image feedback is needed and local expertise is lacking [10]. A growing number of applications have been developed. Some of them, combining clinical, genetic, and laboratory items are able to detect rare or common conditions that would otherwise be missed [10]. ML is divided into three branches, which are selected according to one of the three required research tasks [35,36]: supervised ML for prediction, unsupervised ML for description, and reinforcement learning for causal inference.

Supervised ML (SML) is a predictive model, designed to estimate the likelihood of an event occurring [3]. The predictive analytics applied span from basic computations such as correlation coefficients or risk differences to advanced pattern recognition techniques and supervised learning algorithms like random forests and neural networks, which serve as classifiers or predict the joint distribution of multiple variables [36]. The supervised ML development process involves three subsets of data. First, a training set of labeled data (e.g., histological specimens that have already been labeled as normal or diseased by a human expert) is provided for the algorithm to learn by adjusting weights to minimize the loss of function which calculates the distance between the predicted and true outcome for a given data point [31,35]. Next, the model parameters are optimized using a second validation set [35]. The validation set can also detect overfitting, which is observed when model performance is significantly better on the training set. Finally, a third set is used to evaluate the model’s ability to generalize to new datasets [35]. Once the training session upon labeled data are completed, then the system is applicable to unlabeled data. In this way the trained models predict outcomes through either classification or regression, respectively, in categorical or continuous types of data [31].

Decision trees (DTs) are non-parametric supervised learning algorithms used for classification [37,38]. They map attribute values to classes and have a tree-like structure, including a root node, branches, internal nodes (or decision nodes), and leaf nodes (or terminal nodes) [38,39]. Nodes conduct evaluations, based on the available information, and each node is linked to two or more subtrees or leaf nodes labeled with a class, representing a possible outcome [38,39]. Various types of DTs can be used both in classification and regression tasks [38]. DTs are often preferred over other methods in fields such as healthcare due to their interpretability, despite being less accurate. This is because they are easier to understand and explain compared to other, more complex methods that might be more accurate but relatively uninterpretable [37].

Unsupervised ML (UML) is used for descriptive tasks, with the goal of data clustering and revealing relationships within a data structure [36]. Descriptive tasks provide quantitative summaries of specific features in a certain scenario and require analytics ranging from simple calculations to complex techniques [36]. The main goal of unsupervised learning is to identify inherent groupings or clusters within a data structure, in order to find out data differences, similarities and distributions in feature space [3,29,35]. In unsupervised ML systems, training is data-driven, rather than human-driven, and uses unlabeled data (compared to Supervised ML, whose training features labeled data and is driven by human experts) [36]. This category lacks a guiding response variable during analysis [29].

Reinforcement Learning (RL) is a computational approach where an agent learns to achieve a goal through a trial-and-error cycle in an interactive environment [35]. The agent’s decision-making strategy is improved through its interaction with the environment [40]. The goal of RL is the selection of actions that will maximize future rewards [40]. This is achieved through iterative learning cycles resulting in a reward or penalty in relation to a pre-defined target [31]. For instance, since there is a need for blood glucose concentration monitoring and for an ideal determination of time and amount for insulin delivery in diabetic patients, RL algorithms are potentially capable of learning the individual glucose pattern of a diabetic patient in order to provide adaptive drug supply after a learning process [41]. Changes in the glucose level led to an action of the agent, in terms of insulin injection or no treatment. Subsequently the agent receives a numerical reward, which along with the next glucose level will impact upon the next action [41].

**Figure 1:**
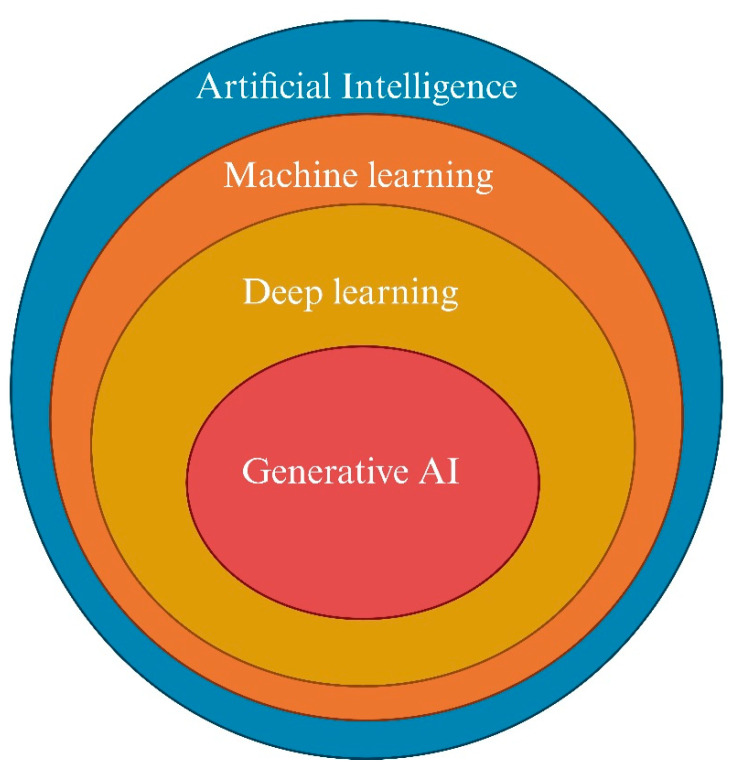
A comparative view of AI, machine learning, deep learning, and generative AI. Created with biorender.com.

**Figure 2:**
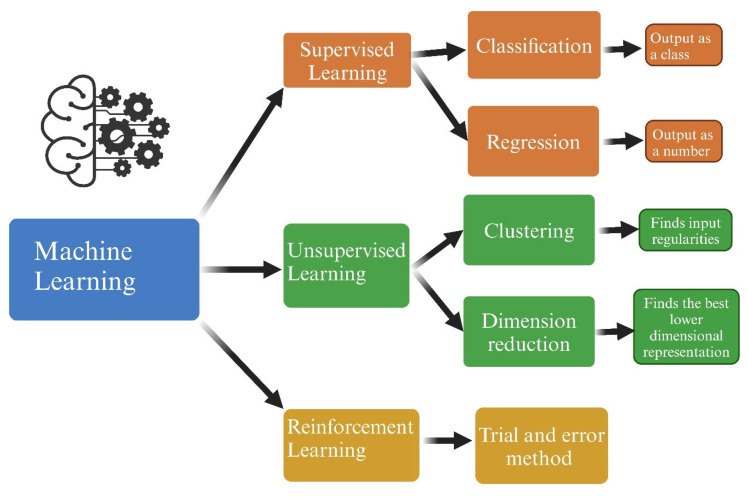
Machine learning algorithms. Created with biorender.com.

AI’s subsets are represented in Figure 1. ML’s subtypes and algorithms are summarized in Figure 2.

Deep Learning (DL) refers to an even more complex subgroup of ML based on numerous processing layers, which may use supervised, unsupervised and reinforcement ML applications [31,42]. In a certain way, it mimics how the human brain builds its own model of the world by learning from large amounts of sensory-motor data acquired through interactions with the environment [40]. DL differs from ML in a number of characteristics. ML requires “manual” feature extraction and processing [43]; it reaches a “plateau” where the quality of performance no longer increases with the volume of data; its training time is somewhat “limited” [35]. On the other hand DL is capable of automatically learning feature hierarchies; it requires a significant amount of data to make predictions, and because it is more computationally intensive than ML, it may require longer training times and state-of-the-art machines to run [31,35]. The complex architecture of DL, consisting of several processing layers that are mostly inaccessible to human users (the so-called “black box of AI”), may pose an issue for the model’s accountability in healthcare [31]. The application of these models is potentially limitless, though not without risk.

Generative AI (GAI) is a type of DL technique that generates realistic facsimiles by evaluating training examples and learning their patterns and distribution [44]. GAI can produce various types of content by using existing sources such as text, audios, images, and videos [44]. One well-known example of its application is ChatGPT, an AI-driven chatbot. Its potential in supporting medical research and clinical practice is currently being assessed [45]. ChatGPT is based on Generative Pre-trained Transformers (GPT—a type of DL model that enables natural language processing) that generate human-like text based on large amounts of data [10,46]. A 2023 systematic review has evaluated its utility in several fields such as data analysis, literature review, scientific writing, medical record storage and management, up to generating diagnoses and management plans [47]. Frequently raised issues concerning generative AI in academic writing include bias, plagiarism, privacy, and legal concerns up to scientific fraud (e.g., fake image synthesis or convincing fraudulent articles resembling genuine scientific papers) [48,49,50]. Therefore the World Association of Medical Editors advises authors and editors to disclose chatbot use in their work and to provide themselves with tools for detecting AI-generated content [31,44]. Furthermore, GAI can also generate non-textual items (images, videos and audios).

Finally when applied to complex analysis of high-dimensional data, including clinical applications, DL can achieve remarkable outcomes [42], e.g., computer-assisted diagnosis of melanoma. In fact, a Deep Convolutional Neural Network trained on images has achieved performances comparable to dermatology experts in identifying keratinocyte carcinomas and malignant melanomas [51].

**Figure 3:**
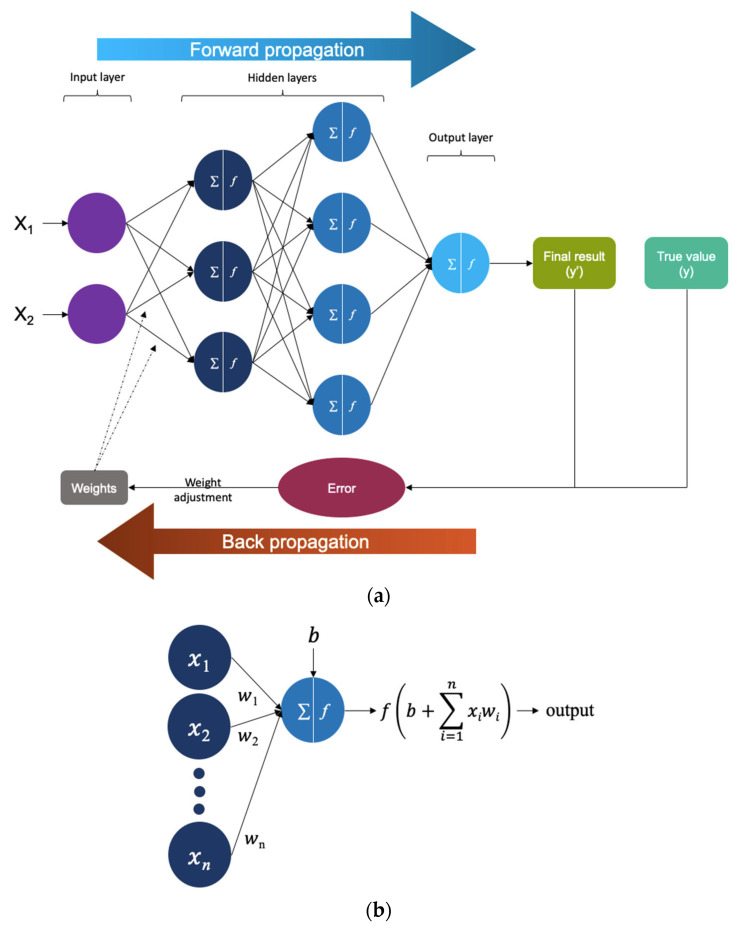
(**a**) Neural networks are the basic architecture of DL models. They are structured in multiple layers consisting of neuron-like interconnected nodes. Data flows through the input layer and into the structure of interconnected nodes in a “forward propagation” mechanism. The final result from the output layer is compared to the true value and a “back propagation” algorithm optimizes results by using prediction error and adjusting weights. (**b**) This is a close-up of a node. The signal received by each node is a result of a weighted linear combination of node outputs from the prior layer, meaning that they are multiplied by a weight assigned to each connection and summed up. A nonlinear transformation is instead applied to the node’s output. *x*_1_–*x*_n_: inputs; *w*_1_–*w*_n_: weights; Σ: summation of weighted outputs from the previous layer; *f*: nonlinear transformation (activation function); and *b*: bias.

Neural Networks (NNs) are the baseline architecture of DL models [52]. They are structured in multiple layers consisting of neuron-like interconnected nodes [35] (Figure 3a,b). Once inserted, data flows along the first layer to the structure of interconnected nodes in a “forward propagation” mechanism [35]. The signal received by each node is a result of a weighted linear combination of node outputs from the prior layer, meaning that they are multiplied by a weight assigned to each connection and summed up. A nonlinear transformation is instead applied to the node’s output [35,52]. The final result from the output layer is compared to the true value and a “back propagation” algorithm optimizes results by using prediction error and adjusting weights [35]. Because NNs are highly parametrized, they might “over-fit” models to data: thus, a series of regularization strategies have been implemented to prevent it [42]. To name one, dropout is a regularization technique where random neurons are dropped, along with their connection, during training [53]. This prevents units from co-adapting too much and helps it to generalize better to unseen data.

All AI models used in clinics and research are summarized in Table 1.

## 4. Current Research and Applications

Clinical Decision Support (CDS) systems can be defined as “computer systems designed to impact clinician decision-making about individual patients at the point in time that these decisions are made” [66]. These systems can be applied in a plethora of medicine fields, including pediatric emergencies. Nevertheless, CDS are not free of limitations, and may sometimes be even perceived as intrusive or ineffective by their users [3]. 

**Figure 4:**
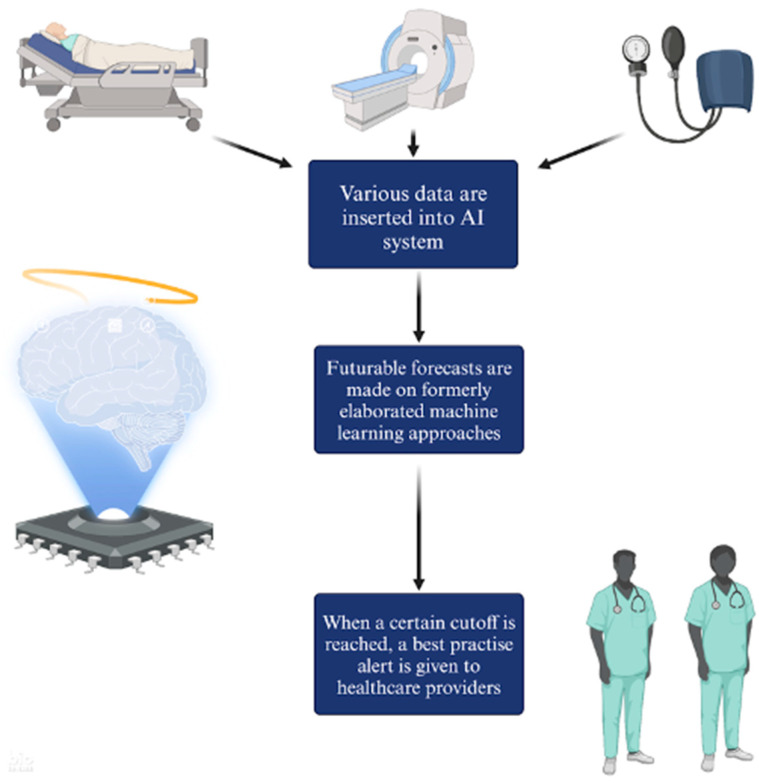
How AI-CDS works. Created with biorender.com.

Compared to traditional rule-based CDS systems, AI-implemented CDS do not rely on statistical algorithms and are occasionally defined as a “non-knowledge”-based CDS [67]. As shown in Figure 4, firstly a wide range of input data are inserted into the AI system. Secondly, this data are used to make predictions. Finally, when a certain threshold is reached, a best practice alert is given to healthcare providers.

### 4.1. AI for Triage Optimization

Triage is the process of quickly assessing sick children when they first present to Pediatric Emergency Departments (PED) in order to classify them into one of the following categories: those with emergency signs who require immediate treatment, those with priority signs who should be given priority in the queue so they can be treated without delay, and those who are non-urgent cases. An efficient triage, performed by healthcare providers, requires expertise but greatly relies on subjective judgement to risk-stratify patients. Several factors such as a first impression of critical care needs and a different subjective pain threshold of patients may affect triage evaluation [68,69,70]. By analyzing the data collected during the triage phase, AI, particularly ML, could be a useful adjunct tool for screening critical patients or those who are candidates for hospitalization [71].

Firstly, in 2018 a multisite, retrospective, cross-sectional study of Emergency Department visits on adults compared an electronic triage system based on a random forest model applied to vital signs, chief complaint, and medical history to standard triage [72]. The electronic triage predictions demonstrated equivalent or improved detection of clinical patient outcomes [72]. 

Subsequently, a retrospective observation cohort study was conducted in Korea in 2019 on a wide range of pediatric patients [73]. They developed prognostic models of critical care and hospitalization through ML, DL, and conventional triage. The predicted variables adopted were the following: age, sex, chief complaint, symptom onset to arrival time, arrival mode, trauma, and vital signs. A DL-based algorithm was developed using a Multilayer Perceptron (MLP) [60] and derivation data, consisting of patient data from January 2014 to June 2016. Test data were then fed into the algorithm, and a risk score between 0 and 1 was obtained, corresponding to the risk of critical care, involving direct admission to the Pediatric Intensive Care Unit (PICU) from the PED or transfer to other hospitals for PICU admission. Two ML models were developed as well for performance comparison, respectively, using logistic regression and random forest. The DL algorithm significantly outperformed the other methods both for intensive care and hospitalization prediction.

In 2022, a prognostic study tested the performance of ML methods in predicting clinical outcomes in children in PED and compared them to conventional approaches [74]. Four learning prediction models were developed: logistic regression with lasso regularization, random forest, gradient-boosted decision tree, and deep neural network. All of them showed better discrimination ability compared to conventional approaches for clinical outcomes, with a higher sensitivity for the critical care outcome and higher specificity for hospitalization [74]. In 2023, Sarty et al. used administrative data collected through triage to train several machine learning models to predict patients who would leave the PED without being seen (LWBS) by an healthcare provider [75]. Among the models applied, XGBoost was the best-performing ML model with 95% recall and 87% sensitivity. The most influential factors in this model were PED patient load, triage hour, minutes taken to drive from the home address to the PED, length of stay, and age. An earlier detection of LWBS would enable a possible development of patient-focused strategies aimed at limiting the phenomenon [75].

### 4.2. AI-Enhanced Socially Assistive Robots for Stress Management

The distressful experiences experimented during healthcare treatments in childhood have been linked to post-traumatic stress disorder and future avoidance of medical care as adults [76]. Stress reduction and compliance maximization are key elements in the management of pediatric patients, especially in emergency settings [77].

Innovative solutions targeting this include AI-enhanced Socially Assistive Robots (SAR), creating a patient-specific support experience aimed at reducing their discomfort during painful procedures such as peripheral intravenous line placement or surgical sutures. SAR systems generally create a relationship without physically touching patients but just through interactions that may include expressiveness, personality, dialog, empathy, and adaptation skills [78].

SARs have already been tested in the PED setting with great results in mitigating children’s discomfort and pain but, to best perform, they need human inputs. They have a limited and scripted behavior and therefore, they lack a tailored and flexible feedback in this unpredictable emergency background [76,79]. Nevertheless, it is pivotal to acknowledge that the aim of the SAR is not to replace human involvement in mitigating distress and pain management, but to add a tool that healthcare providers may use.

Two clinical research articles collected concerns and expectations to implement the design of an AI-enhanced SAR [80,81]. Healthcare providers felt that the proposed SAR should be equipped with a wide range of skills to meet children’s needs, involving encouraging dialogue, positive reinforcement expressions, humor, and cognitive behavioral strategies (e.g., breathing techniques and meditation) [81]. Caregivers saw the primary function of the SAR as a distractor during painful procedures. Specifically, they suggested behaviors including tricks, jokes, playing music, singing, and dancing [80].

### 4.3. AI for Traumatic Brain Injury Assessment

Head trauma is one of the leading causes of PED admissions worldwide [82]. Since the majority of them are constituted by minor traumas, it is essential to make a straight distinction to ensure a prompt management when needed [83]. A CT (Computed Tomography) scan is considered the gold standard examination to detect a brain injury but it has several drawbacks: it is expensive, may eventually require sedation and boosts the risk of cancer in a lifetime due to radiations exposure [84]. A set of rules, known as the PECARN rules, has been validated to risk-stratify this category of patients to find out which patients ought to undergo a radiological imaging [85].

In recent years, AI approaches have been applied to the diagnosis and management of traumatic brain injury in pediatric patients [86,87].

In 2017 Dayan et al. implemented the PECARN rules with a multi-faced intervention, focused on computerized CDS, to provide the bedside pediatrician with directive indications for CT use and supporting data (e.g., risk estimates for TBI) [88]. CDS components involved: determination of whether the patient matched the age-specific PECARN very low risk criteria; a recommendation that CT was not indicated if the child met the very low risk criteria; risk estimations for clinically relevant TBI; and links to the prediction rule principles and paper. They showed this intervention was associated with modest, safe, but variable decreases in CT use without missing any relevant brain damages [88]. 

In 2018 Hale et al. developed an Artificial Neural Network (ANN) trained on clinical items and radiologist-interpreted imaging findings to identify patients at risk for clinically relevant TBI [89]. A total of 12,902 pediatric patients were enrolled in this study from the PECARN TBI dataset. Trough the elaboration of clinical and radiological data ANN showed 99.73% sensitivity with 98.19% precision, 97.98% accuracy, 91.23% negative predictive value, 0.0027% false negative rate, and 60.47% specificity for clinically relevant TBI [89].

In 2019, Bertsimas et al. compared an approach based on optimal classification tree (OCT) with original PECARN rules in Traumatic Brain Injury (TBI) management, examining the same sample of children previously treated with PECARN rules [90]. OCTs are classification trees similar to the classification and regression trees (CARTs) that were used to derive the original PECARN rules but fitted with a novel method (Mixed Integer Optimization) that could outperform the classical CART-fitting algorithms [37]. Outcomes suggested that OCTs performed as well as or better than PECARN rules in identifying children at very low risk of clinically important traumatic brain injury [90]; thus, the potential application of OCTs may provide a valuable tool to decrease unnecessary CT scans while maintaining adequate sensitivity for identifying patients with clinically significant TBI.

In 2023, Miyagawa et al. used a decision tree method to predict the necessity of CT scans in children under 2 years of age with mild TBI. This kind of SML achieved this outcome with a rate of 95% accuracy [91]. Focusing on the contribution of each predictor on the decision tree, days of life was the most significant. According to these findings, days of life could be used as a main factor for decision making for head trauma in children younger than 2 years of age, and could substitute age in years in clinical flowcharts [91].

Nowadays a significant proportion of pediatric neuroimaging performed attempts to generate the outcome prediction of brain injury as either hypoxic and traumatic [92,93]. Subtle mild TBI anomalies not visualized on CT or on conventional MRI (Magnetic Resonance Imaging) can be detected through state-of-the-art neuroimaging. One of the most significant among them is diffusion MRI, which enables qualitative and quantitative assessment of specific white matter tracts in the nervous system [94]. The study of the network of white nerve fibers, known as the “connectome”, has recently received increasing attention [95]. Connectome mapping using post-processing methods through diffusion MRI-based fiber tracking, such as track density imaging and edge density imaging, is a new frontier in research because it can reveal abnormalities even in mild TBI, such as white matter damage not seen on CT and MRI [96]. Raji et al. used Support Vector Machines (SVMs) to analyze patients with TBI based on edge density imaging [97]. SVMs examine and group labelled data into classes, split by the widest plane (support vector). They are often employed when there is a non-linear correlation among data, and, as such, a separation line is not easily recognizable [98]. In their study, Raji et al. identified three white matter regions distinguishing mild TBI from controls using edge density imaging maps. Bilateral tapetum, sagittal stratum and callosal splenium identified mild TBI subjects with sensitivity of 79% and specificity of 100%; accuracy from the area under the receiver operating characteristic (ROC) curve (AUC) was 94%. In this study, edge density imaging could provide better diagnostic delineation of pediatric mild TBI than neurocognitive assessment of memory or attention [97].

### 4.4. AI for Pediatric Sepsis Prediction

Sepsis is the main cause of death worldwide in pediatric patients resulting in an estimated 7.5 million deaths annually [99]. Sepsis is a life-threatening organ dysfunction associated with infection. Prompt and accurate identification of sepsis requires data-driven screening tools with affordable precision and high sensitivity [100]. Several scores for sepsis identification have been designed over years, even though they often lack in sensibility and specificity [101,102].

Systemic Inflammatory Response Syndrome (SIRS) criteria were previously included in the pediatric sepsis definition by Goldstein et al. but they had poor predictive properties [103]. 

In 2024, the Society of Critical Care Medicine task force suggested that sepsis in children is defined by a Phoenix Sepsis Score of at least two points in children with suspected infection, meaning potentially life-threatening disfunction of respiratory, cardiovascular, coagulation, or neurological systems [100]. Even though the Phoenix sepsis criteria performed well, future independent validation is needed, especially in low-resources settings. In recent years computerized sepsis prediction systems have been developed to overcome intrinsic limitations of pediatric sepsis scores. By accessing electronic health record (EHR) data for clinical decision support, these systems can detect early septic patients whose treatment would otherwise be delayed [104]. In 2018, Kamaleswaran et al. conducted an observational cohort study. They analyzed continuous minute-by-minute physiological data of 493 PICU patients over a timeline of 24 h to assess the onset of severe sepsis [105]. Twenty of this cluster of patients developed severe sepsis. The authors demonstrated that AI could identify patients with severe sepsis before they clinically show relevant findings by just assessing quantifiable physiomarkers, such as heart rate (HR), mean blood pressure (MBP), systolic blood pressure (SBP), diastolic blood pressure (DBP), and oxygen saturation (SpO_2_). Furthermore, these algorithms were able to detect severe sepsis 8 h earlier than a currently implemented real-time electronic screening tool in critically ill children. These findings pointed out how is pivotal for bedside monitors to be combined with artificial intelligence to improve their predictivity of severe sepsis. 

In 2019 Le et al. tested a ML-based prediction algorithm using EHR [106]. The ML system adopted was based upon boosted ensembles of decision trees. Ensemble classifiers sum up the output from weak apprentices, each of which would be inadequate to solve the problem autonomously, generating a more efficient learner. Every single baseline learner in this paper was a decision tree. Each tree was constructed by repeatedly splitting the feature space, acquiring thresholds within the features which most reduce entropy, and therefore enhance information [106]. Their algorithm outperformed in terms of sensibility, specificity and accuracy the Pediatric Logistic Organ Dysfunction score (PELOD-2) (*p* < 0.05) and the pediatric Systemic Inflammatory Response Syndrome (SIRS) score (*p* < 0.05) in the prediction of severe sepsis [106].

In 2022, Stella et al. used a different approach which focused on predicting the need of resuscitation within 6 h of triage, rather than diagnosing sepsis [107]. In this way, the model could provide actionable decisional support. Data were extracted from EHC and involved demographics, triage vitals, triage nurse comments, chief complaint information, as well as orders placed, and medications administered within 6 h of arrival. Several models were employed including standard and regularized regression, random forests, gradient boosted trees and generalized additive [107]. Moving the aim from the diagnosis of severe sepsis to the provision of resuscitative care allowed the development of severe sepsis to be avoided, rather than treating the full blown condition [107].

In 2023, Mercurio et al. conducted a retrospective observational study of children presenting to a PED at a tertiary care children’s hospital with fever, hypotension, or an infectious disease International Classification of Diseases (ICD)-10 diagnosis [108]. They proved that combining clinical and sociodemographic variables, sensibility and specificity performance of ML methods were as high as 93% and 84%, respectively, in identifying patients with diagnosis of sepsis. The random forest classifier performed the best, followed by a classification and regression tree [108]. The maximum recorded heart rate and the maximum mean arterial pressure resulted in the two most significant factors in determining the model. Other unexpected variables such as age, immunization status and demographics data have proved relevant in the early detection of sepsis as well [108].

### 4.5. Challenges and Future Perspectives

Accomplishing accuracy is an issue for AI in healthcare. Physician expertise and behavior are unsuitable items to be replaced by AI systems [109]. In addition, challenges such as the inability to critically capture anatomical, physiological, and pathological details hinder the roadmap to a robust and trustworthy AI healthcare domain [110]. Furthermore, inconsistencies between different input sources can ultimately lead to unreliable outputs from AI systems. This can undermine the trustworthiness of AI. Parents’ and patients’ trust in AI tools is a factor that should not be underestimated. As a matter of fact a recent study showed a moderately open parental attitude toward the use of AI-driven healthcare interventions in their child’s medical care [111]. Sisk et al. identified seven major parental concerns: quality/accuracy, privacy, shared decision making, convenience, cost, human element of care, and social justice [111]. In particular, opacity and lack of transparency in AI decision-making processes could strain the relationship between parents and pediatricians. The lack of real-world analysis through a visual representation is a major concern, as it allows for a lack of assessment and interpretation. In fact, evidence generated by AI is often referred to as inscrutable due to varying degrees of opacity in how the data are processed and interpreted [112]. 

Two other alerts about the quality of AI-generated evidence emerge: inconclusive and misguided evidence. Outcomes obtained through a machine learning process produce, as with any process of analysis, probable knowledge and so, are not infallible. A statistical analysis could provide some items to prove a correlation but, in many cases, this is not sufficient [113]. Similarly, evidence generated by AI ought to be critically interpreted and revised to provide insight into the decision-making process and to avoid misleading results [114]. Moreover, the difficulty of ensuring fairness and equity in AI-generated recommendations is highlighted by the presence of bias, inversion, and redundancy [115]. In this context, responsible AI is an emerging field. It involves the moral accountability of state-of-the-art technologies [116]. Its main aim is to establish ethical principles that can minimize bias and promote equity, simplify the readability of processes, and ultimately enhance the robustness and safety of outcomes [117].

Another issue is how AI systems reshape datasets, selecting or excluding information that might be relevant; this could be a breach of privacy by limiting patients’ access to data and their ability to understand how recommendations are derived from the data [118].

In this review we focused on the aforementioned challenges that AI should face in order to provide insightful support for medical decision making, but we highlighted future research directions as well. As we are consistently experimenting an increase of PED overcrowding, waiting times and length of stay, the current limitations of triage practice and the massive amount of healthcare data point out the need for a smarter triage assessment in emergency settings [119]. In relation to this, the present literature shows that machine learning algorithms allow the automation of processes that can speed up extracting significant relationships from datasets to predict outcomes [119]. ML-based triage models have proven to be superior to standard triage in the prediction of critical outcomes and hospital admissions [120]. They have shown to be able to find out meaningful patient symptoms, analyze patients’ medical history, and forecast patient needs based on the accessible EHRs [120]. Even though these models obtained good prediction outcomes, there is room for improvement for future research perspectives. Previous studies are mainly based on retrospective data, collected during PED visits. Future trials ought to focus on validating new models using prospective data with a robust sample size enrollment. Currently, studies in this research area focus on comparing AI and standard triage performances rather than improving the triage model and patient flow processes within the hospital environment [121]. As a matter of fact, an efficient triage is not a stand-alone compartment but is closely linked to hospital dynamics, infrastructures, consumable supplies, and human resources [122]. Future researchers ought to take all these factors into account to train a model on large data samples from multiple sources to enhance the generalization and the performance within a specific hospital setting.

As we previously suggested, AI, through different models, may provide a valuable tool to decrease unnecessary CT scans while maintaining adequate sensitivity for identifying pediatric patients with clinically significant TBI [90]. Connectome mapping using post-processing methods with diffusion MRI-based fiber tracking, such as track density imaging and edge density imaging, is a new frontier in research because it can reveal abnormalities even in mild TBI that are not observed on CT and MRI. This imaging has particular importance to mild TBI due to the related white matter damage that appears at a microstructural level because of rotational, stretching and shearing forces [123]. Such imaging techniques, suitably integrated and analyzed with ML methods, can potentially provide objective quantitative biomarkers for the diagnosis, and prognostication of children with mild TBI. Future studies ought to focus on a longitudinal diffusion MRI evaluations and on validating the previously described imaging patterns to predict patient outcomes [97]. Utilizing larger sample sizes and direct assessment of specific brain domains can also help in achieving a deeper understanding of how neural circuitry differences relate to individual patient injury and outcome [97].

**Figure 5:**
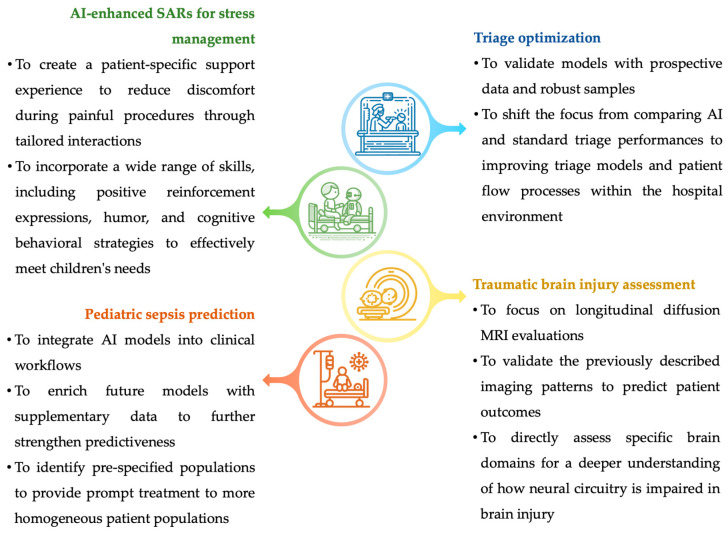
Future feasible research perspectives. AI: Artificial intelligence. MRI: Magnetic Resonance Imaging. SAR: Socially Assistive Robot.

**Figure 6:**
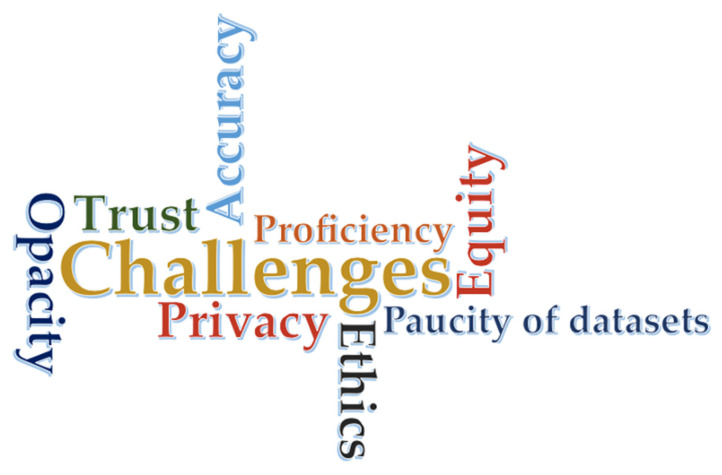
Challenges of AI in pediatric emergency medicine.

Due to the burden of morbidity and mortality caused by pediatric sepsis and the importance of starting a prompt treatment, there is a need for effective predictive risk models to allow physicians to identify patients at high risk in order to intervene as soon as possible [108]. In recent years a bunch of computerized sepsis prediction models have been developed and have outperformed pediatric sepsis scores in most cases [106,107]. Future research in this area is needed to plan applicable strategies for incorporating these models into the clinical workflow. Furthermore, most models use only a small amount of data available to pediatricians, which may represent a chance to enrich future models to further strengthen predictiveness. To provide prompt treatment to more homogeneous patient populations, machine learning models could be explored to accurately identify pre-specified populations [124]. Future feasible research perspectives and challenges are illustrated, respectively, in Figure 5 and Figure 6.

## 5. Discussion

Although further validation is required, AI might represent a useful supporting tool for PED decision-making and has the potential to improve the timely allocation of resources and interventions [74]. In the literature, some previous studies have addressed our same topics. In 2018, Liu et al. explored a wide range of applications of AI in emergency medicine, both in adults and pediatric patients. They subcategorized the applications into three main areas: predictive modelling, patient monitoring, and emergency department operations. In the early years of application of AI in medicine, they emphasized how insightful its contribution could be in different emergency instances [125]. Afterwards, in 2022, Alzhani et al. conducted a narrative review asserting AI and machine learning tools have been proven as reliable accompaniment of pediatricians [126]. Their key findings partially overlap with ours. Specifically, they found that AI is a valuable support in terms of early diagnosis of pediatric septic shock, prediction of disease severity in traumatic brain injury, and emergency resource management. The main barriers to a widespread diffusion they identified are the lack of structured data based on large samples of the pediatric population, as well as legal and trust issues. Our review took a more comprehensive approach than these previous studies, focusing more on the real challenges and limitations to a widespread adoption, until future research prospects. In the first part, we explored the theoretical principles of AI, providing all the necessary background to feel confident with these new state-of-the-art tools. The second part presents an informative analysis of AI models in pediatric emergencies. For each of them, we focused on the results achieved over the years, highlighting current obstacles and potential areas of improvement. Our analysis shows that in order to make progress towards a widespread use, it is essential to tailor AI algorithms to specific clinical needs and to integrate algorithms into clinical workflows. This requires a close partnership between PED clinicians and developers. Recognizing and addressing a series of barriers is crucial for a safe development of efficient AI tools [127,128]. The first key item is ensuring AI an accurate input. Expert clinicians ought to select features to be accurately included in datasets for ML training because inaccurate training datasets lead to suboptimal diagnostic accuracy [129]. ML subsequent iterations may amplify these errors, reinforcing biases introduced in the early phases [35]. In most cases monocentric datasets fail to correctly address the heterogeneity of pediatric conditions, particularly in the Emergency Department. Pediatric populations accessing different PEDs may differ heavily, particularly when comparing rural and urban PEDs, leading to significant biases in the training phase. Failure to account for this can lead to misdiagnosis [130]. Additionally, the peculiarities of the emergency-urgency network of a certain region may lead to some centers being the reference hubs for a subset of pathologies (e.g., neurological disorders), leading to an overrepresentation of certain conditions in the dataset. 

These datasets should be the result of a multidisciplinary team of professionals working towards a predefined goal. As a notorious quote says: “garbage in—garbage out”, meaning input datasets are crucial in determining the final outcome [131]. Ensuring data quality requires a dedicated infrastructure, i.e., patient health records, diagnostic images, and real-time data monitoring. Relying excessively on paper-based systems and having operator-built databases introduces several potential pitfalls for error, potentially reducing the final accuracy and reproducibility of the algorithms [132]. Confirmation bias is another item to be considered. AI recognizes patterns on which it has trained upon but it is unlikely to identify what it is not taught [133]. Further relevance should be given to ensuring AI models are explainable and their outputs can be justified and critically assessed by physicians and healthcare professionals in a transition from opaque “black box” models to more transparent “(translucent) glass box” models, enabling physicians and healthcare professionals to critically assess and justify the outputs [134]. This is particularly crucial in emergency settings, where the accuracy and reliability of AI systems are of utmost importance. In such high-stakes scenarios, the decision-making process of AI models must be clear and easily interpretable to ensure swift and appropriate medical interventions. To achieve this level of explainability, various approaches can be employed, such as transparent modeling techniques or post-hoc explanations. Transparent modeling involves inherently interpretable models, while post-hoc explanations provide insights into the model’s reasoning after the fact. The choice between these approaches depends on the specific healthcare application and the balance between explainability and model performance.

In addition, ensuring external validation is vital for quality control. When applied to an external dataset, a certain clinical support tool may not produce sensible or specific results [93]. Additionally, the algorithm’s performance can quickly reduce as clinical practices dynamically evolve. Hence, a continuous influx of data are pivotal to refine the model and keep it current [35]. Some of these limitations are particularly true for pediatrics. Children stand only for a marginal portion of healthcare resources and their datasets are frequently small [3]. Granular data (i.e., displaying a high level of detail in the way data are structured), ideal for ML applications, are rarely available in pediatrics. Targeting a right balance between granularity and simplicity is a key factor in optimizing AI performance and ensuring significant outcomes from complex datasets [135]. Furthermore, children data are not homogenously distributed resulting in inequality, with some data sharply prevailing on others due to significative variations in features according to the patient’s age. AI models ought to account for variations and changes in disease risk that occur according to age [136]. Vital and auxological parameters have to be interpreted according to age as well. Even radiologic images need to be interpreted taking into account the age and the resulting changes in anatomy, physiopathology and possible differential diagnosis. All the previously mentioned issues could be challenging in validating an automated analysis [137]. 

The absence of evidence-based and variability in care are other factors limiting the application of AI in pediatrics. In fact, a wide range of pediatric conditions has no gold standard care universally shared and treatment significantly varies among different institutions [138,139]. 

The integration of AI and ML into pediatric emergency wards faces the same barriers as other technologies integrated into clinical practice [140] and demands a workforce that is proficient in these technologies. Healthcare professionals must be upskilled or reskilled to understand the capabilities and limitations of AI and ML, interpret the outputs of these systems, and integrate this information into clinical decision-making processes [141]. Training programs and continuous education initiatives are essential to equip healthcare professionals with the knowledge and skills needed to work alongside AI and ML technologies effectively [142]. This not only enhances the quality of patient care but also ensures that healthcare professionals can remain competitive and adapt to the evolving landscape of healthcare technology.

Most of the studies retrieved by our review were conducted outside of the European Union. This can be partly linked to the attention that AI-assisted software and medical devices receive in the EU, e.g., compared to the United States. The EU’s recently approved AI Act [143] categorizes AI as “high risk” when it is implemented in health and care; this wary approach is not far from what was previously observed in the General Data Protection Regulation and similar norms, which impose stricter regulations on the use of health data for research and training purposes [144]. Most of the studies we reviewed were conducted in the U.S.A., whose Food and Drug Administration has issued several documents defining AI and ML software as “medical devices” [145], providing additional guidance on good practice to develop them. A recent scoping review suggested similar results on the broader field of clinical trials [146]. 

Finally, AI poses ethical questions, especially when it comes to liability. Will pediatricians be responsible for eventual consequences that do not fit predictions? At the present time, clinicians may be liable for harm to patients if they observe AI indications to use nonstandard care methods. Current law only protects doctors from liability when they follow the standard care. Nevertheless, as AI becomes integrated in gold standards, we could speculate physicians would probably avoid liability when following AI indications [147].

## 6. Conclusions

AI offers many promises in pediatric emergency with a wide range of applications involving triage optimization, predictive models for traumatic brain injury assessment, and computerized sepsis prediction systems. In each of these areas, AI models outperformed standard methods. In a few years, AI could integrally reshape how we approach pediatric emergencies. The main barriers to a widespread adoption include technological challenges, but also ethical issues, age-related differences in data interpretation, and the paucity of comprehensive datasets in the pediatric context. Future feasible research directions should address the validation of models through prospective datasets with more numerous sample sizes of patients. Furthermore, our analysis shows that it is essential to integrate models into the clinical workflow and tailor AI algorithms to specific medical needs. This requires a close partnership between PED clinicians and AI developers. Building a shared knowledge platform is therefore a key step. Overall, the next phases aim to bridge the current gap between research and clinical practice, striking a delicate balance between the transformative potential of AI and its current limitations.

## Figures and Tables

**Table 1:** AI models used in clinics and research.

System	Description
Artificial Neural Network (ANN)	Nodes, akin to neurons, process information, while connections between layers, termed edges, simulate synapses with weights. Output is computed via mathematical operations on input and hidden layers, with the learning algorithm adjusting weights to minimize errors between predicted and target outputs, forming probability-weighted associations stored within the network’s structure [54].
Backpropagation Neural Network	Backpropagation utilizes prediction errors to iteratively tune the weights, enabling the NN to learn patterns within the training data and enhance model accuracy over time [35].
Convolutional Neural Network (CNN)	CNNs process data that comes in the form of multiple arrays such as signals, images, audio spectrograms and videos, and is applied in the recognition of objects [52].
Deep Neural Network (DNN)	An ANN with numerous layers between the input and output layers which is capable of learning high-level features and requires high computational power [55].
Probabilistic Neural Network (PNN)	An application of DNN within probabilistic models, able to capture complex non-linear stochastic relationships between random variables [56].
Recurrent Neural Network (RNN)	A recurrent neural network (RNN) is any network whose neurons send feedback signals to each other, and are capable of modeling sequential data for sequence recognition and prediction [57,58].
Region-based Convolutional Neural Network (R-CNN)	R-CNN models use region-based networks, which are capable of detecting an object in an image and holds great potential especially in diagnostic imaging [59].
Multilayer Perceptron (MLP)	A feedforward type of powerful and dynamic ANN. The signals are transmitted within the network in one direction: from input to output [60].
Bayesian Inference	Bayesian statistical methods are applied to algorithms. They start with existing ‘prior’ beliefs, which are then updated using data to give ‘posterior’ beliefs, which may be used as the basis for inferential decisions [61].
Causal Associational Network (CASNET)	Three items constitute this model: patient observation, pathophysiological states, and disease classifications. Once documented, the observations are associated with the fitting states [62].
Light Gradient Boosting Machine (LightGBM)	LightGBM employs a boosting strategy to combine numerous decision trees, with each tree utilizing the negative gradient of the loss function as the residual approximation for fitting. It is designed for optimal performance, particularly in distributed systems [63].
Extreme Gradient Boosting (XGBoost)	XGBoost is a gradient boosting framework that is highly efficient and scalable. It features a proficient linear model solver and a tree learning algorithm. It enables diverse objective functions, such as regression, classification, and ranking. Its design allows for easy extension, enabling users to define custom objectives [64].
Natural Language Processing (NLP)	NLP is a subfield of AI and ML used to interpret linguistic data (e.g., clinical note analysis and decision making) [10,42].
Random Forest Models	Random forest models use randomization to create multiple decision trees, each contributing to the final output. In classification tasks, the trees’ outputs are combined through voting, while in regression tasks, they are averaged to produce a single output [65].
